# Direct observation of catalytic oxidation of particulate matter using *in situ* TEM

**DOI:** 10.1038/srep10161

**Published:** 2015-07-08

**Authors:** Kohei Kamatani, Kimitaka Higuchi, Yuta Yamamoto, Shigeo Arai, Nobuo Tanaka, Masaru Ogura

**Affiliations:** 1 Institute of Industrial Science, The University of Tokyo, Komaba 4-6-1, Meguro-ku, Tokyo 153-8505, Japan; 2 Ecotopia Science Institute and Graduate School of Engineering, Nagoya University, Chikusa-ku, Nagoya 464-8603, Japan; 3 Unit of Elements Strategy Initiative for Catalysts & Batteries, Kyoto University, Katsura, Nishikyo, Kyoto 615-8510, Japan

## Abstract

The ability to observe chemical reactions at the molecular level convincingly demonstrates the physical and chemical phenomena occurring throughout a reaction mechanism. Videos obtained through *in situ* transmission electron microscopy (TEM) revealed the oxidation of catalytic soot under practical reaction conditions. Carbon oxidation reactions using Ag/SiO_2_ or Cs_2_CO_3_/nepheline catalysts were performed at 330 °C under an O_2_ flow of 0.5 Pa in the TEM measurement chamber. Ag/SiO_2_ catalyzed the reaction at the interface of the mobile Ag species and carbon, while the Cs species was fixed on the nepheline surface during the reaction. In the latter case, carbon particles moved, remained attached to the Cs_2_CO_3_/nepheline surface, and were consumed at the interface by the oxidation reaction. Using this technique, we were able to visualize such mobile and immobile catalysis according to different mechanisms.

Recently, diesel-engine automobiles have been attracting significant attention relative to gasoline-engine automobiles as they emit relatively low amounts of carbon dioxide (CO_2_) per unit driving distance. However, the particulate matter (PM) that is released with the diesel exhaust is harmful to the environment and human health^1^. To reduce the PM emitted to the surrounding environment, a diesel particulate filter (DPF) can be installed, and it is currently the most widely-accepted approach for trapping the PM. Regeneration of the DPF is frequently required to remove PM accumulation for minimizing pressure loss.

The emitted PM is mainly comprised of carbonaceous soot. The oxidation of PM by gaseous oxygen occurs at around 600 °C, which cannot be accomplished by the diesel exhaust, approximately 200 °C–400 °C^2^. Generally, the filtered PM is oxidized by increasing the exhaust temperature either via combustion of additional fuel or addition of external energy such as an electric source, resulting in decreased fuel efficiency. Physical damage to the DPF structure by the high temperature has also been reported^3^; therefore, an alternative solution for DPF regeneration at lower temperatures would be advantageous.

The oxidation of PM at a decreased temperature using a catalyst has been investigated. Oxygen and nitrogen dioxide already present in the exhaust can be utilized as oxidizing reagents for carbonaceous materials. Although NO_2_ has a higher oxidizing reactivity, it is generated only on platinum-group catalysts via NO oxidation at low temperatures in wet atmosphere. Therefore, the diesel oxidation catalyst would require a large quantity of Pt metal. Furthermore, the concentration of O_2_ in diesel exhaust is thousand times higher than that of NO_2_^4^. Therefore, O_2_ is the preferred oxidizing agent for PM oxidation.

Currently, effective catalysts for carbon oxidation by O_2_ are alkali metal oxides^5^,^6^,^7^, Ag^8^,^9^, CeO_2_-based mixed oxides^10^,^11^,^12^,^13^, and perovskites^14^,^15^. These catalysts are classified into two categories: “mobile catalyst” or “mobile oxygen”^16^,^17^. The mobile catalyst, including metal and alkali compounds, has a low melting point. In the reaction atmosphere, the catalyst undergoes solid–liquid phase transformation, leading to an increase in the contact area of the liquefied catalyst with the reactant (PM). Catalysts involving mobile oxygen species such as CeO_2_ show high intrinsic activity for PM oxidation, as the active mobile oxygen is easily produced at the catalytic surface/interface with carbon from inside the bulk oxide.

Catalytic PM oxidation occurs under solid (PM, carbon)–solid (catalyst)–gas (O_2_) triphasic conditions when using mobile species. Unlike other catalytic reactions under heterogeneous conditions (e.g., gas reactant–solid catalyst or liquid reactant–solid catalyst), the solid–solid–gas triphasic reaction proceeds with some difficulty depending on the contact between the two solids. The contact between the PM and catalyst is crucial in promoting the reaction^18^. However, this triphasic reaction and its mechanism in relation to a solid–solid reaction are still unclear; few investigations have been conducted toward a detailed analysis of this process. A catalytic reaction mechanism has been speculated on the basis of the information about the surface before and after the reaction. Real-time investigation of the reaction mechanism on solid catalysts under operating conditions and in the presence of reactants at high temperatures remains a challenge; however, recent studies have used *in situ* and *operando* analyses (XRD^19^, IR^20^. To understand the practical reaction mechanism, we focus on *in situ* analysis by TEM through which we can observe and investigate the actual surface in real time under operating conditions. Several experiments using *in situ* TEM have been reported: observation of catalytic reactions with gas and solid catalysts^21^, crystal growth processes^22^,^23^, and investigations of supported metal behavior (e.g., melting point)^24^,^25^. Herein, we assume a very simple reaction for PM oxidation on solid catalyst, C_(s)_+O_2(g)_→CO_2(g)_, where the reaction is triphasic. We report *in situ* observation of catalytic PM oxidation using TEM and demonstrate how PM oxidation proceeds, as well as catalysts’ behavior during oxidation.

## Experimental

We initially focused on alkali carbonate catalysts loaded on nepheline, an aluminosilicate that showed high tolerance for hydration and leaching of alkali species^26^,^27^,^28^,^29^. Furthermore, Ag catalysts have been reported and recognized worldwide to show high performance for PM oxidation. In this study, Cs_2_CO_3_-loaded nepheline and Ag supported on SiO_2_ were chosen as representative mobile catalysts. Here we have chosen cesium compound rather than potassium or sodium ones because the former showed higher catalytic performance than the latter ones^29^, meaning that lower temperature is expected to be required for the TEM analysis. Also, cesium could be analyzed more clearly than potassium or sodium because of the difference in their atomic weights from the other components such as aluminosilicate nepheline and carbon.

Silica-supported Ag catalyst containing 10 wt% of Ag was prepared with the incipient wetness method using an aqueous AgNO_3_ solution (Wako Pure Chemical) and powder SiO_2_ (fumed, Aldrich). After heating till complete evaporation of the aqueous phase, the precipitated product was calcined at 500 °C for 3 h in air.

The nepheline-loaded Cs_2_CO_3_ catalyst was prepared as follows. A Na form Lind-type A zeolite with Si/Al molar ratio of 1 (Wako Pure Chemical) was sintered at 1000 °C for 4 h in air to give Na-nepheline through solid–solid phase transformation^26^,^27^,^28^. Cs_2_CO_3_ (Wako Pure Chemical) was then loaded on the Na-nepheline support by impregnation (10 wt%). This was immersed in distilled water, and the solvent was evaporated at 100 °C under vigorous stirring. The precipitated product was calcined at 800 °C for 3 h in air.

The catalytic activity test for PM oxidation was performed using temperature-programmed oxidation on a thermogravimetric–differential thermal analyzer (TG-DTA, Rigaku Thermo Plus TG8120). Carbon black #2600 (Mitsubishi Chemical) of diameter 13 nm was used as the reference for PM particles. The prepared catalyst and carbon were mixed in an agate motor for 10 min, generally called a “tight contact”. The catalytic activity test was normally conducted by two types of contacts; one is “loose contact”, for which the soot is mixed with the catalyst by using a spatula, and the other is “tight contact”, for which the soot and catalyst are mixed in an agate motor. It has long been recognized that the “tight-contact” scenario is more reflective of the intrinsic catalytic performance. Catalyst/carbon weight ratio was set at 10. The mixture was then heated to 800 °C at 10 K min^−1^ under a flow of dried air. The catalytic activity for the carbon oxidation was evaluated by the temperature T_ig_, the point at which a distinct exothermic DTA curve derived from the heat of the carbon oxidation starts to appear.

Carbon oxidation on a Cu-microgrid was observed at an accelerating voltage of 1 MeV, under 0.5 Pa O_2_ atmosphere at 330 °C on a TEM (JEM-1000K RS; JEOL). A small amount of the tight-contact-mode catalyst and carbon was placed on the Cu-microgrid. This was placed in the sample holder, which equips with the furnace and temperature controller, and inserted in the TEM. First, O_2_ gas was introduced into the environmental cell chamber by gradually increasing the pressure surrounding the Cu-microgrid to 0.5 Pa, and then heated to 330 °C. Details of the image recording system for the JEM-1000K RS TEM are described in a previous study^30^.

## Results and Discussion

**Figure 1:**
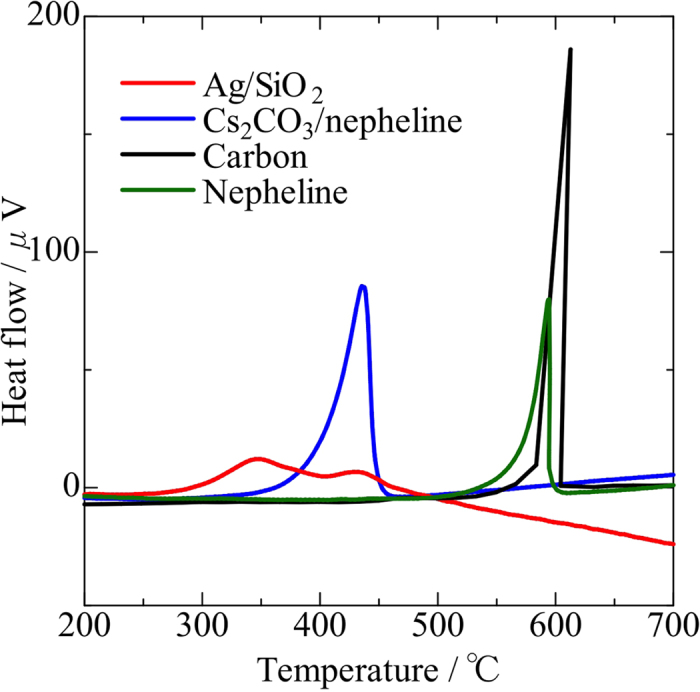
Catalytic performance of carbon oxidation. Ag/SiO_2_ (red line), Cs_2_CO_3_/nepheline (blue line), and nepheline (green line). The black line is carbon oxidation without catalyst (noncontact oxidation by gaseous oxygen).

The catalytic performance of carbon oxidation is shown in Fig. 1. Nepheline and SiO_2_ (not shown in the figure) showed next to no catalytic activity for the oxidation because their DTA curves closely resemble those of the noncatalytic reaction of carbon with gaseous oxygen. The carbon oxidation using Ag/SiO_2_ and Cs_2_CO_3_/nepheline occurred at 255 °C and 311 °C, respectively, indicating that the Ag catalyst shows a higher oxidation activity than the Cs_2_CO_3_ catalyst.

**Figure 2:**
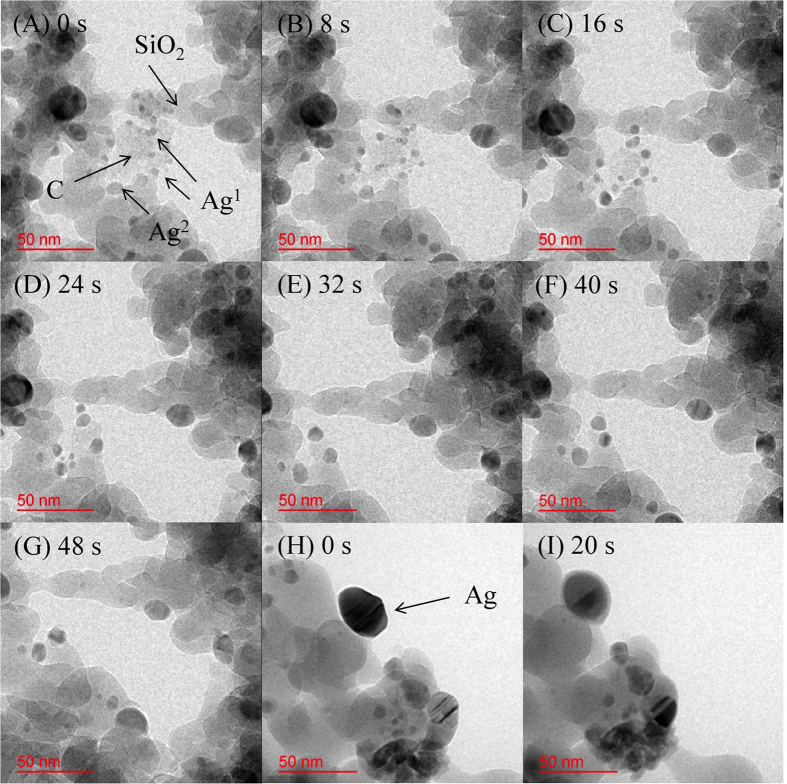
*In situ* TEM images of the Ag/SiO_2_ catalyst. (**A**)–(**G**) show the time course images for carbon oxidation using Ag/SiO_2_ at 330 °C with an O_2_ gas flow of 0.5 Pa. (Ag^1^: mobile, Ag^2^: silent) TEM images (**H**) and (**I**) show another position for the same Ag/SiO_2_ without carbon under the same conditions.

Figures 2 and SI_2 display a series of TEM images obtained during the carbon oxidation using Ag/SiO_2_ at 330 °C with a 0.5 Pa O_2_ supply. Images (A)–(G) were acquired over 48 s, with one image captured every 8 s. As shown in image (A), Ag particles can be observed as black dots with their diameters ranging from 5 to 25 nm. During the carbon oxidation, two Ag species were observed: *mobile* Ag particles on SiO_2_ (Ag^1^ in (A)) and *silent* Ag particles on SiO_2_ (Ag^2^ in (A)). The C–O–Ag interaction may be slightly stronger than the Si–O–Ag one. The *mobile* Ag on SiO_2_ transferred to carbon particles, and oxidation appeared to occur at the active interface of Ag and carbon. As the reaction proceeded, the *mobile* Ag agglomerated into larger particles. Finally, the *mobile*, agglomerated Ag terminated the reaction by becoming *silent*, without any contact with the carbon around the Ag particle. In the case of the *silent* Ag, from the first reaction stage, the carbon particles around Ag seemed to transfer to the active interface while maintaining contact with Ag during the reaction. To identify the reason for Ag mobility, the behavior of Ag/SiO_2_ without the carbon was investigated. By comparing the TEM images before (H) and after (I) the reaction, the morphology and interface/surface of the Ag particle was changed from a crystal-like faceted plane to a spherical shape. Furthermore, the contrast of the Ag particles in image (I) decreased from the surface to the inside, indicating that the particle surface melted and became spherical because of the surface tension.

These results implied that liquefaction may be occurring at the Ag/SiO_2_ interface according to the definition of Tammann temperature^31^. Surface premelting of Ag began at 550 °C–650 °C in vacuum depending on the Ag particle size^32^. Coalescence of Ag particles was also observed at 150 °C^33^. In this study, during the carbon oxidation, Ag particles on carbon easily agglomerated, which might be due to the heat of the carbon oxidation at the interface and partial liquefaction of the Ag surface at 330 °C. The mobile range of Ag transferred from the SiO_2_ surface onto the carbon particles became limited as carbon was consumed. Consequently, we can deduce that Ag/SiO_2_ can be used as a *mobile* catalyst for PM oxidation.

**Figure 3:**
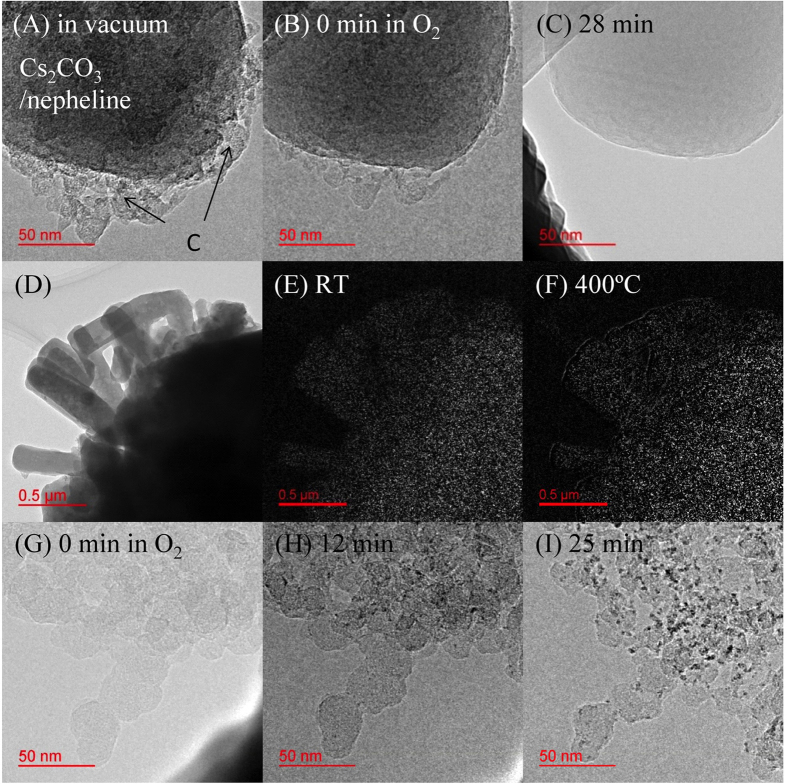
*In situ* TEM images of Cs_2_CO_3_/nepheline catalyst. (**A**)–(**C**) show TEM time course images for carbon oxidation using Cs_2_CO_3_/nepheline at 330 °C with an O_2_ gas flow of 0.5 Pa. (**D**) shows the TEM image of Cs_2_CO_3_/nepheline, and (**E**) and (**F**) show EELS mapping on Cs_2_CO_3_/nepheline for Cs *M*-core at RT and 400 °C, respectively. (**G**)–(**I**) show TEM time course images during carbon oxidation using only Cs_2_CO_3_ at 330 °C with an O_2_ gas flow of 0.5 Pa.

Figures 3(A) and SI_3 show the TEM image of Cs_2_CO_3_/nepheline physically mixed with carbon at 330 °C in vacuum. Images (B) and (C) demonstrate the time course of carbon oxidation at 330 °C, with O_2_ flowing at 0.5 Pa, acquired over 28 min. In image (A), the dark spheres correspond to Cs_2_CO_3_/nepheline, and the gray shadows are the agglomerated carbon particles. As explained above, the nepheline itself did not catalyze carbon oxidation at 330 °C; therefore, the oxidation activity derives from the Cs species. Images (A)–(C) led us to conclude that carbon oxidation occurs at the whole surface of the Cs_2_CO_3_/nepheline. This also indicates that the Cs species was highly dispersed on the nepheline surface. During the reaction, carbon particles continuously moved toward the Cs_2_CO_3_/nepheline surface, where carbon was oxidized.

Image (D) shows a TEM image of Cs_2_CO_3_/nepheline, and images (E) and (F) display electron energy loss spectroscopy (EELS) mapping images of the Cs *M*-core at RT and 400 °C, respectively. The Cs species on nepheline was in a high dispersion state at RT, which was maintained even at 400 °C, revealing a specific interaction between the Cs species and nepheline. Images (G)–(I) summarize the TEM time course during the carbon oxidation using Cs_2_CO_3_ at 330 °C with an O_2_ flow of 0.5 Pa. Black dots derived from Cs cationic species became distinct as carbon oxidation proceeded. Notably, such black dots did not appear on the carbon in the TEM images (A)–(C). This result also indicates the specific interaction between the Cs cationic species and negatively-charged nepheline surface (Coulomb attraction). Alkali metal catalysts are currently categorized as *mobile* catalysts under PM oxidation conditions owing to their low melting point. However, this study clearly suggests that the Cs species on nepheline maintains its high dispersion state and is *silent* on the support during the carbon oxidation. Such high stability of alkali metals on nepheline has been demonstrated in our previous study^26^,^27^,^28^. Thus, we emphasize that the properties of the supported metals significantly depend on the nature of, and interaction with, the substrate. Therefore, it is evident that Cs_2_CO_3_/nepheline has potential application as a *mobile oxygen* catalyst for PM oxidation.

Note that the TEM analyses of the PM oxidation performance investigated herein are not simply minor phenomena occurring within the very limited field of view being observed. As indicated in the Supplementary Materials, we compared the kinetics observed by the TEM views and detected by the TG-DTA measurements, and the reaction rate of the carbon oxidation under the same conditions, which showed a comparable order, suggesting that the views by TEM can be recognized as the intrinsic performance of the catalysts reported in this study.

## Conclusions

*In situ* TEM was successfully applied for the elucidation of the PM oxidation mechanism for two catalyst types: Ag/SiO_2_ and Cs_2_CO_3_/nepheline. The Ag particles were *mobile* during the carbon oxidation at 330 °C in an O_2_ atmosphere at 0.5 Pa. The carbon was consumed by the *mobile* Ag particles, causing the Ag particles to agglomerate. The reaction was finalized when all the carbon was consumed from the Ag–C islands. The Cs species were fixed *silently* on nepheline during the reaction even at 400 °C, which was indicated via EELS mapping. Interestingly, the interface of carbon and nepheline particles was continuously generated where carbon was oxidized. Finally, we show how, by visualizing these reactions at the TEM level, we were able to not only demonstrate the catalytic mechanism but also explain some physical and chemical characteristics of these catalysts. Seeing is not only believing, but also realizing and seizing an actual phenomenon in catalysis.

## Additional Information

**How to cite this article**: Kamatani, K. *et al.* Direct observation of catalytic oxidation of particulate matter using *in situ* TEM. *Sci. Rep.* **5**, 10161; doi: 10.1038/srep10161 (2015).

## Supplementary Material

Supplementary Information

Supplementary Movie S1

Supplementary Movie S2
