# Supplementary material for: Direct observation of catalytic oxidation of particulate matter using in situ TEM
Source: Sci Rep. 2015 Jul 8;5:10161. doi: 10.1038/srep10161 (PMC4495604; doi:10.1038/srep10161)
Supplement: Supplementary Information [file srep10161-s1.docx]

**Title: Direct observation of catalytic oxidation of particulate matter using *in situ* TEM**

**Authors:** Kohei Kamatani^1^, Kimitaka Higuchi^2^, Yuta Yamamoto^2^, Shigeo Arai^2^, Nobuo Tanaka^2^, and Masaru Ogura^1,3*^

**Supplementary Materials:**

SI_1. Validity of *in situ* TEM analysis

*In situ* TEM observations were performed at 330°C with O_2_ flow at 0.5 Pa. Observation of the catalytic carbon oxidation using *in situ* TEM provides specific information that can lead to an inaccurate representation of the bulk material. To avoid this, we confirmed the results obtained by TEM and TG-DTA analyses.

The reaction rate of carbon oxidation was calculated from the TEM analyses in Figs. 3 (**B**) and (**C**). The carbon particles used in this study were assumed to be spherical, with a diameter of 13 nm and density of 1.70 g·cm^−3^. For images (**B**) and (**C**), 1.05 × 10^−20^ g of carbon per second was oxidized and consumed. The contact area with the Cs_2_CO_3_/nepheline catalyst was estimated (from the image) to be 63.9 nm in length and 13 nm in depth, resulting in 8.31 × 10^−16^ m^2^. Therefore, the reaction rate was calculated to be 1.26 × 10^−5^ g-carbon·m^−2^-catalyst·sec^−1^. The TG-DTA analysis was performed under isothermic conditions at 330°C for 28 min to correspond to the time period between Figs. 3 (**B**) and (**C**). Consumption of the weight loss of carbon by oxidation was 1.72 × 10^−7^ g-carbon·sec^−1^. The carbon was recognized in a highly dispersed state, completely on the Cs_2_CO_3_/nepheline under the tight contact mode, and the oxidation proceeded uniformly over the whole Cs_2_CO_3_/nepheline surface, which was given by the Brunauer–Emmett–Teller surface area (m^2^·g^−1^-cat) and the weight determined by the TG analysis (2.2 × 10^−2^ m^2^-cat). Finally, the TG-DTA analysis determined that the reaction rate was 7.8 × 10^−6^ g-carbon·m^−2^-cat·sec^−1^. Hence, we concluded that the *in situ* TEM analyses reported are a representative of the intrinsic catalytic performance for carbon oxidation at increased temperatures.

SI_2. *In situ* TEM video (double-speed) of Ag/SiO_2_ during carbon oxidation

SI_3. *In situ* TEM video (octuple-speed) of Cs_2_CO_3_/nepheline during carbon oxidation
